# Supplementary material for: A prebiotic basis for ATP as the universal energy currency
Source: PLoS Biol. 2022 Oct 4;20(10):e3001437. doi: 10.1371/journal.pbio.3001437 (PMC9531788; doi:10.1371/journal.pbio.3001437)
Supplement: S1 Results — (DOCX) [file pbio.3001437.s001.docx]

Supplementary Information Results

A prebiotic basis for ATP as the universal energy currency

Silvana Pinna^1^, Cäcilia Kunz^1^, Aaron Halpern^1^, Stuart A. Harrison^1^, Sean F. Jordan^1^, John Ward^2^, Finn Werner^3^ and Nick Lane^1*^.

Silvana Pinna ORCID: 0000-0002-3680-1219

Aaron Halpern ORCID: 0000-0002-0105-7354

Sean F. Jordan ORCID: 0000-000108403-1100

Stuart A. Harrison ORCID: 0000-000205329-7747

John Ward ORCID: 0000-0002-4415-5544

Finn Werner ORCID: 0000-000203930-3821

Nick Lane ORCID: 0000-0002-5433-3973

*^1^Centre for Life’s Origins and Evolution (CLOE), Department of Genetics, Evolution and Environment, University College London, Darwin Building, London, United Kingdom*

*^2^Department of Biochemical Engineering University College London, London, United Kingdom*

*^3^Institute for Structural and Molecular Biology, University College London, Darwin Building, London, United Kingdom*

^*^nick.lane@ucl.ac.uk


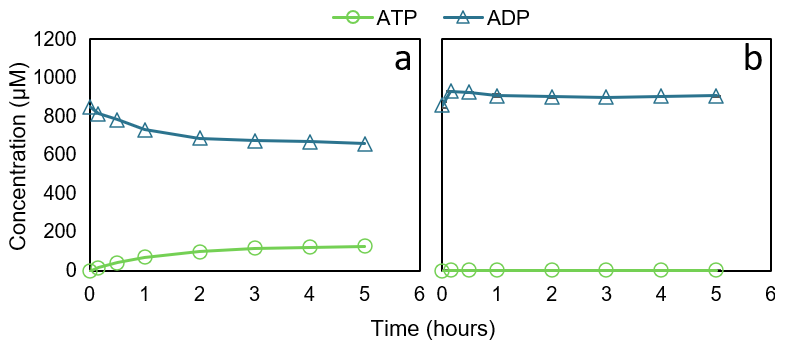


**Fig A** – ATP synthesis in the presence or absence of AcP. (a) The reaction ADP (1 mM) + AcP (4 mM) + Fe^3+^ (0.5 mM) and (b) the control ADP (1 mM) + Fe^3+^ (0.5 mM), at 30 °C and pH ~5-6. N = 3 ±SD. The data underlying this Figure can be found in Table Z and AA in S1 Data (sheet 7).


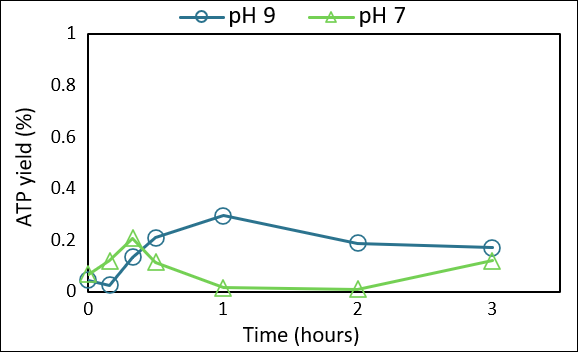


**Fig B** – Comparison of the ATP yield from the reaction ADP (1 mM) + AcP (4 mM) at 30°C in a FeS clusters-rich 10 mM bicarbonate solution at pH 9 (circles, teal) and 7 (triangles, green). The data underlying this Figure can be found in Table AB in S1 Data (sheet 8).


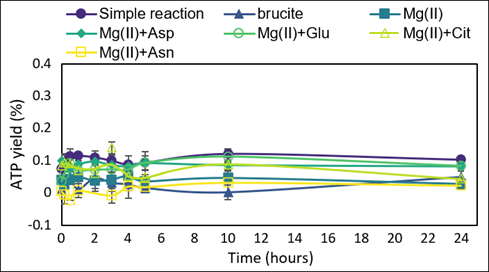


**Fig C** – Comparison of reaction ADP (1 mM) + AcP (4 mM) at 30°C and pH ~5.5–6 with different forms of magnesium (ionic form and mineral form brucite) coordinated by citrate or amino acids. N = 3 ±SD. Brucite is a hydroxide mineral (Mg(OH)_2_) with a unit structure reminiscent of the Mg^2+^ coordination by aspartate in enzymes such as Mg^2+^-dependent RNA polymerase. The data underlying this Figure can be found in Table AC and AD in S1 Data (sheet 9).

*Panel a*


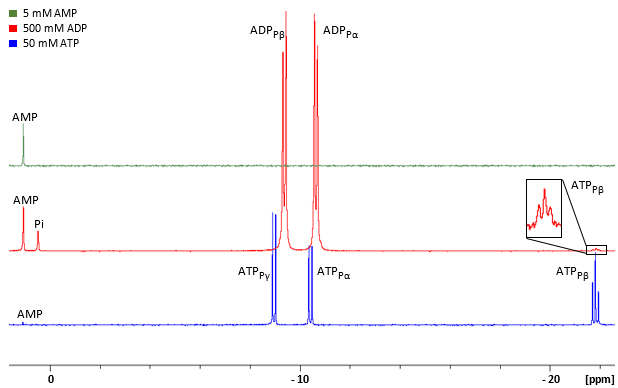


*Panel b*


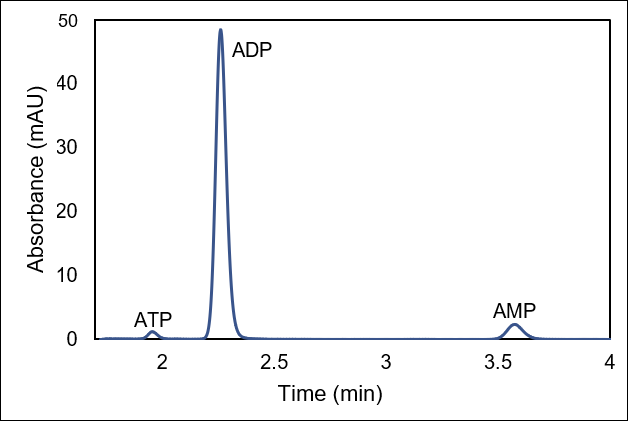


**Fig D –** Residual levels of ATP present in the ADP commercial standard. (a) Comparison of ^31^P-NMR spectra of commercial AMP (green), commercial ADP (red), and commercial ATP (blue); the graph insert shows a zoomed in area of ATP signal. (b) HPLC chromatogram of commercial ADP (1mM). All peaks labelled for clarity. The data underlying this Figure can be found in Figure B and Table AE in S1 Data (sheet 10).


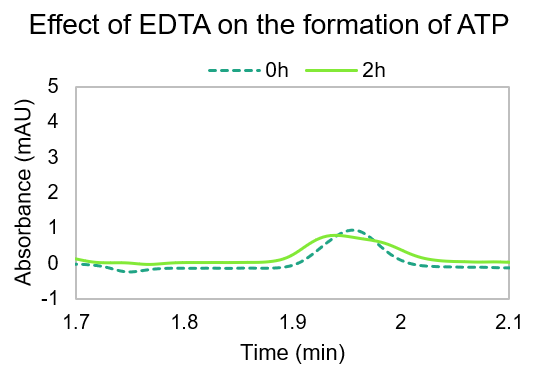


**Fig E –** HPLC chromatogram of the reaction ADP (1 mM) + AcP (4 mM) + Fe^3+^ (0.5 mM) + EDTA (500 µM) at the beginning of the reaction (0 h, broken line) and after 2 hours (2 h, solid line), at 30 °C and pH ~5-6 (zoomed-in view of the ATP peak). The widening of the peak is due to a problem in the HPLC column, but it does not affect detection. The data underlying this Figure can be found in Table AF and AG in S1 Data (sheet 11).

**Fig F –** Possible stacking of ADP coordinated by Fe^3+^.

**
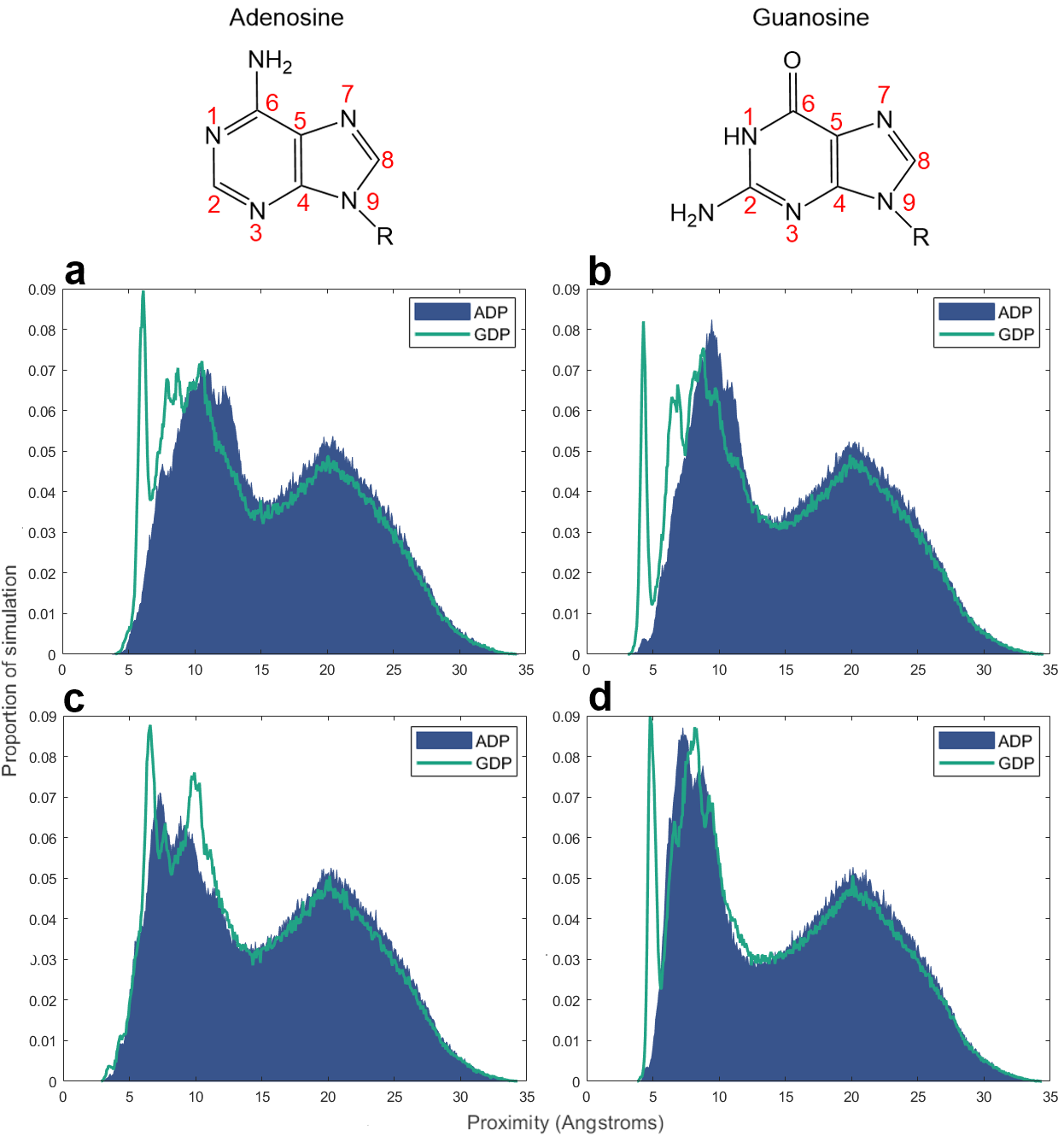
**

**Fig G –** Proximity of ferric iron to different ring nitrogens in ADP or GDP in a simulation containing 8 AcP, 2 Fe^3+^, 8 Li^+^, 4 Na^+^, 12 K^+^, 18 Cl^-^and either 2 ADP or 2 GDP. The ADP, GDP and AcP have protonation states of –2-.  **a.**  Proximity of ferric iron to N1 **b.** Proximity of ferric iron to N3 **c.** Proximity of ferric iron to N7 **d.** Proximity of ferric iron to N9. The data underlying this Figure can be found in Table AH – AK in S1 Data (sheet 12).


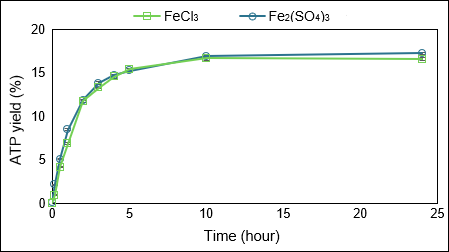


**Fig H –** Comparison of the ATP yield from the reaction ADP (1 mM) + AcP (4 mM) + Fe^3+^ (0.5 mM) at 30°C where the Fe^3+^ is given by either FeCl_3_ (squares, green) or Fe_2_(SO_4_)_3_ (circles, teal). N = 3 ±SD. The data underlying this Figure can be found in Table AL in S1 Data (sheet 13).

*Numerical data is available in the Excel file S1 Data*
