# Supplementary material for: A prebiotic basis for ATP as the universal energy currency
Source: PLoS Biol. 2022 Oct 4;20(10):e3001437. doi: 10.1371/journal.pbio.3001437 (PMC9531788; doi:10.1371/journal.pbio.3001437)
Supplement: Attachment — Submitted filename: The manuscript by Pinna et al.pdf [file pbio.3001437.s003.pdf]

The manuscript by Pinna et al. reports on the detailed characterization of the phosphorylation of ADP by acetyl phosphate (AcP) to form ATP in the presence of ferric ions. This previously observed reaction appears to be surprisingly specific as no other NDPs or transition metal ions can carry out equivalent reactions to any significant extent. ADP phosphorylation appears to be optimal at 30°C, mildly acidic pH and low salt. The authors provide explanations for their observations within the context of their already well-established hypothesis of life's origins in a hydrothermal vent. Besides the fact that, taken face value, the observations reported here would rather favor a surface pond or lake as the cradle of life, some of their propositions need to be further substantiated.

Page 3 "ATP in a prebiotic, monomeric world". This assumes that life originated in bulk water. But, if, instead, life first appeared on mineral surfaces then the needed energy could have been provided through the oxidation of H_2 and CO by, for instance, catalytic FeNiS minerals. Only when more evolved protocells liberated themselves from those surfaces, and moved to bulk water, would have ATP become essential.

"Even if only one nucleotide triphosphate can be dominant, the implication of a frozen accident is not a satisfying explanation." Why not? There are different levels of "frozen accidents". This has been discussed by Brandon Carter who, in 1984, proposed that the evolution towards intelligent life required 6 "hard steps", the first one being abiogenesis. Each step was statistically postulated to last between 600 and 800 million years. Although these periods of time may be considered workable in astronomy and geology, in biology they do not make the same sense. In the latter case it is necessary to invoke the occurrence of extremely unlikely (highly contingent) events to explain that time duration. They can be indeed defined as "frozen accidents".

Page 13. "Such steep pH gradients could in principle operate across protocells as well as inorganic barriers." The authors propose that ATP would have been synthesized outside protocells, under acidic conditions, to be then transported across a proto-membrane into a cell where, under alkaline conditions, it would have phosphorylated some substrate(s). Have they considered the implications of such a mechanism in terms of ATP/ADP concentrations and diffusion? ATP would have to diffuse towards the protocell and ADP do it in the opposite direction, without risking to disappear into the bulk water. Another crucial point is membrane permeability. As F. H. Westheimer wrote in 1987: "Phosphoric acid is specially adapted for its role in nucleic acids because it can link two nucleotides and still ionize; the resulting negative charge serves both to stabilize the diesters against hydrolysis and to retain the molecules within a lipid membrane. A similar explanation for stability and retention also holds for phosphates that are intermediary metabolites and for phosphates that serve as energy sources." So, one very important role of even proto-membranes should have been to keep charged metabolites inside the cell to sustain metabolism. This, in turn, would have made ADP diffusion out of the protocell difficult to envision.

Page 15. "…enabling ATP to drive work even in a prebiotic monomer world." How are the authors defining 'work' in that setting? In contemporary biology work is i) performed mechanically as in muscle contraction and ii) against a gradient of chemical potential in active transport (see Jencks, 1989). This work depends on complex protein-based binding energy modulation and the coupling of intermediate reaction steps. Of course, this kind of work could not have existed prebiotically. At that time the most likely role of ATP (if it already existed) would have been , for instance, phosphorylation to render carbonyl carbons electrophilic

enough to be attacked by a nucleophile such as ammonia. It is debatable that this process would qualify as work.

Page 31. Figure 6. Here and in the text, it is implied that the N6 -NH_2 group interacts with Fe^{3+}. In A-T base pairing N6 interacts with the O atom from the carbonyl group of T through a polarized δ+ proton. What would be the driving force in its postulated interaction with Fe^{3+}? In **b**, the metal ion is hexacoordinated but the coordination geometry is not clear. In any case, it doesn't look octahedral. Maybe the authors should do some modeling to check whether the proposed arrangement is plausible. An experimental confirmation of their model would be even better.
