# Supplementary material for: A prebiotic basis for ATP as the universal energy currency
Source: PLoS Biol. 2022 Oct 4;20(10):e3001437. doi: 10.1371/journal.pbio.3001437 (PMC9531788; doi:10.1371/journal.pbio.3001437)
Supplement: Attachment — Submitted filename: Pinna et al PLOS Biol response to reviewers.pdf [file pbio.3001437.s004.pdf]

DEPARTMENT OF GENETICS, EVOLUTION AND ENVIRONMENT
UNIVERSITY COLLEGE LONDON

A prebiotic basis for ATP as the universal energy currency

Silvana Pinna[1], Cäcilia Kunz[1], Aaron Halpern[1], Stuart A. Harrison[1], Sean F. Jordan[1], John Ward[2], Finn Werner[3] and Nick Lane[1*].

We have addressed the comments of the reviewers and the Academic Editor in detail below. The reviewers' comments are in Roman text and our answers are in italics. We thank the all four referees for their enormous time, effort and expertise in reviewing our paper.

REVIEWERS' COMMENTS:

Reviewer #1:

The manuscript by Pinna et al. reports on the detailed characterization of the phosphorylation of ADP by acetyl phosphate (AcP) to form ATP in the presence of ferric ions. This previously observed reaction appears to be surprisingly specific as no other NDPs or transition metal ions can carry out equivalent reactions to any significant extent. ADP phosphorylation appears to be optimal at 30°C, mildly acidic pH and low salt. The authors provide explanations for their observations within the context of their already well-established hypothesis of life's origins in a hydrothermal vent. Besides the fact that, taken face value, the observations reported here would rather favor a surface pond or lake as the cradle of life, some of their propositions need to be further substantiated.

We thank R1 for this clear synopsis. We agree that taken at face value our observations might seem to favour freshwater environments and we make this clearer in the revised version (p14 lines 401-408). We have also revised the order of the Discussion to bring up this passage before the section on work, as the paragraphs on work may have obscured this point in the original version.

Page 3 "ATP in a prebiotic, monomeric world". This assumes that life originated in bulk water. But, if, instead, life first appeared on mineral surfaces then the needed energy could have been provided through the oxidation of H2 and CO by, for instance, catalytic FeNiS minerals. Only when more evolved

****
Prof Nick Lane
Professor of Evolutionary Biochemistry
Department of Genetics, Evolution and Environment
University College London
Room 610 Darwin Building
Gower Street, London WC1E 6BT

External phone +44 (0) 20 7679 1385
Internal phone 31385
nick.lane@ucl.ac.uk
www.nick-lane.net

protocells liberated themselves from those surfaces, and moved to bulk water, would have ATP become essential.

We have revised the Introduction to make our reasoning clear here. We agree with R1 that the earliest forms of metabolism are indeed exergonic and could have occurred on mineral surfaces. But it is unlikely that this could account for purine nucleotide synthesis, for example, which has six phosphorylation steps. We lay out this perspective in the revised Introduction, specifically on pages 3-4, lines 69-83.

"Even if only one nucleotide triphosphate can be dominant, the implication of a frozen accident is not a satisfying explanation." Why not? There are different levels of "frozen accidents". This has been discussed by Brandon Carter who, in 1984, proposed that the evolution towards intelligent life required 6 "hard steps", the first one being abiogenesis. Each step was statistically postulated to last between 600 and 800 million years. Although these periods of time may be considered workable in astronomy and geology, in biology they do not make the same sense. In the latter case it is necessary to invoke the occurrence of extremely unlikely (highly contingent) events to explain that time duration. They can be indeed defined as "frozen accidents".

We do not agree with R1 about frozen accidents, but this is a relatively trivial point in relation to the paper. We have de-emphasised frozen accidents in the revised Introduction, and instead stressed the centrality of other adenine nucleotide cofactors including NAD, FAD and coenzyme A, all of which point to the importance (and so availability) of adenosine or ATP in early metabolism.

Page 13. "Such steep pH gradients could in principle operate across protocells as well as inorganic barriers." The authors propose that ATP would have been synthesized outside protocells, under acidic conditions, to be then transported across a proto-membrane into a cell where, under alkaline conditions, it would have phosphorylated some substrate(s). Have they considered the implications of such a mechanism in terms of ATP/ADP concentrations and diffusion? ATP would have to diffuse towards the protocell and ADP do it in the opposite direction, without risking to disappear into the bulk water. Another crucial point is membrane permeability. As F. H. Westheimer wrote in 1987: "Phosphoric acid is specially adapted for its role in nucleic acids because it can link two nucleotides and still ionize; the resulting negative charge serves both to stabilize the diesters against hydrolysis and to retain the molecules within a lipid membrane. A similar explanation for stability and retention also holds for phosphates that are intermediary metabolites and for phosphates that serve as energy sources." So, one very important role of even proto-membranes should have been to keep charged metabolites inside the cell to sustain metabolism. This, in turn, would have made ADP diffusion out of the protocell difficult to envision.

This is a complete misunderstanding of what we had attempted to write, and we can only apologize for not being sufficiently clear. We agree with R1 (and Westheimer's classic paper) about phosphorylated intermediates, including especially triphosphates, not being able to cross cell membranes, even simple fatty acid bilayers. In fact, we envisaged ADP being phosphorylated inside the protocells immediately adjacent to the membrane, subject to proton influx from the acidic exterior. No phosphates ever cross the membrane in our model. We have made this clear in the revised Discussion (page 15, lines 435-443).

Page 15. "…enabling ATP to drive work even in a prebiotic monomer world." How are the authors defining 'work' in that setting? In contemporary biology work is i) performed mechanically as in muscle contraction and ii) against a gradient of chemical potential in active transport (see Jencks, 1989). This work depends on complex protein-based binding energy modulation and the coupling of intermediate reaction steps. Of course, this kind of work could not have existed prebiotically. At that time the most likely role of ATP (if it already existed) would have been , for instance, phosphorylation to render carbonyl carbons electrophilic enough to be attacked by a nucleophile such as ammonia. It is debatable that this process would qualify as work.

Our perspective is grounded in the idea of a monomer world, and we do not envisage any protein-based machinery here at all. We have made this clearer at several points in the revised MS and added a new paragraph to the end of the Discussion (page 15 lines 451- 464) where we discuss specifically what we mean by work in this context (essentially coupling exergonic to endergonic reactions, and in particular the polymerization of nucleotides and amino acids).

Page 31. Figure 6. Here and in the text, it is implied that the N6 -NH2 group interacts with Fe^{3+}. In A-T base pairing N6 interacts with the O atom from the carbonyl group of T through a polarized delta+ proton. What would be the driving force in its postulated interaction with Fe^{3+}? In b, the metal ion is hexacoordinated but the coordination geometry is not clear. In any case, it doesn't look octahedral. Maybe the authors should do some modeling to check whether the proposed arrangement is plausible. An experimental confirmation of their model would be even better.

We thank R1 for pointing out that base pairing in DNA and agree that our model as originally depicted was incorrect. We have also undertaken a series of molecular dynamic simulations, as suggested by R1, which do indeed strengthen our mechanistic interpretations. We discuss these new MD simulations at length in the Results section on page 10, lines 259-288, in two new figures (Fig. 6 and SI Fig. 7), and in the Discussion on page 12 lines 330-337. We use this new information to modify our proposed mechanism, specifically in relation to the N6 amine group and the N7, which is presented in Fig. 7. These new sections also respond to the concerns of other reviewers.

We thank R1 again for their helpful comments and hope that we have addressed them to their satisfaction. The revisions very clearly improve the paper.

Reviewer #2:

Pinna et al. present a study on the prebiotic formation of ATP from the Fe^{3+} catalysed reaction between ADP and acetyl phosphate. The authors attempt to answer a very important question in prebiotic chemistry which is how did ATP become the principle energy currency in Life and, in particular, why did ATP become dominant over the other nucleotide triphosphates (NTPs) for this purpose.

The authors extend the work of Kitani (Ref 32 and 33) who first demonstrate that ATP could be formed from a reaction between acetyl phosphate (AcP) and ADP in the presence of Fe^{3+} ions. The authors explore whether other metal ions can also catalyse this reaction but find that Fe^{3+} is unique in its ability to do so. The authors further explore the effect of pH, temperature, salt solutes of varying concentration and pressure on the reaction. The authors repeat the pH experiments carried out by Kitani (Ref 33) which affirm the reaction proceeds optimally at mildly acidic pHs. The temperature optimum was at 30

oC. Higher salt concentrations significantly affected the reaction and pressure had a negligible effect. The authors then proceed to test different prebiotically plausible phosphorylating reagents to see if there are others apart from AcP that can perform the reaction. The authors find that carbamoyl phosphate (CP) is the only other phosphorylating reagent which works.

Next the authors explore whether Fe^{3+} catalysed phosphorylation of ADP by AcP also works for other NTPs. The authors find that the reaction exclusively phosphorylates ADP. Finally the authors attempt to unravel the mechanism behind why only ADP is phosphorylated. The authors attempt the phosphorylation of pyrophosphate to determine whether the nucleobase is important and conclude that it is. The authors conclude that a purine ring is important due to the lack of phosphorylation of the pyrimidines. Of the three purine rings explored only adenosine showed phosphorylation and therefore the authors conclude that the N6 amine is essential as both guanosine and inosine have a carbonyl group at the 6-position on the purine ring. The authors then determine that the optimal stoichiometry for ADP and Fe^{3+} is 1:1. Following Kitani they also find that addition of Mg^{2+} or Ca^{2+} increases the yield of the reaction. Lastly the authors conduct a MALDI-ToF experiment to ascertain whether

Fe^{3+}-ADP complex stacking is present in the reaction and conclude that it isn't. This all leads to propose the mechanism in Figure 6 whereby a macro chelate complex is formed between a single Fe^{3+} and a single ADP and the adenosine coordinates to the Fe^{3+} via the N6-amine and the pyrophosphate. AcP then coordinates to the Fe^{3+} and the phosphate is transferred and then Mg^{2+} displaces Fe^{3+} to promote catalyst turnover. Given that only ADP is phosphorylated to ATP the authors therefore conclude that this is the reason why Life uses ATP as the universal energy currency and that this has its origins in prebiotic chemistry.

We thank R2 for their highly detailed and extremely helpful review, which we have addressed in detail, to the substantial improvement of the paper. We are very grateful for the time and expertise that went into this invaluable review. The paragraphs above are a very clear and accurate synopsis.

I consider that the key result in this paper, namely that only ATP is phosphorylated and not the other NTPs, to be a very interesting result and this will be of great interest to the Origins of Life community. The paper is overall well written and well referenced. The experiments up to and including the demonstration that only ADP is phosphorylated by the reaction have been performed well, although I believe an additional control experiment is required to prove that ADP is being phosphorylated only by AcP in the presence of Fe^{3+}. However, I have a number of serious concerns with both the experiments used to probe the mechanism of the reaction (the 31P NMR spectroscopy and the MALDI ToF experiments) and with the mechanistic interpretation of the results from these experiments which run contrary to what is known in the literature. The authors need to address these concerns in detail. Furthermore, the final conclusion of the paper where the authors claim that they have solved the reason why ATP is the universal energy currency of Life is not fully substantiated by the results presented in the paper. The authors have significantly overstretched themselves in making this claim and they need to tone down the conclusion of the paper substantially. I detail my reasoning behind this assessment in comments below.

We thank R2 for the generally very constructive tenor of their review and for their specific comments. In brief here (as we address them in more detail later): the missing control had indeed been done, but had not been included in the previous MS. It is now included as SI Fig. 1 and does duly show that Fe^{3+} with ADP does not catalyse its disproportionation to AMP and ATP. We accept R2's concerns about the NMR

work and decided to drop it from the paper altogether as it was also an issue for other reviewers and did not add much if anything to the paper. However, we cannot agree that our MALDI-ToF work is not useful. We have previously detected stacks of AMP using the same methodology and the same instrument, so we do not doubt that it can detect stacks. Moreover, the new MD simulations support our interpretation, so we have bolstered the argument here. In deference to the expertise of R2, we have nonetheless been careful to couch our argument in more tentative terms. As noted in response to R1, we have modified our mechanistic interpretation along the lines suggested by R2 but now supported by the MD simulations; however we do not agree that ADP dimers play a role in phosphorylation. Finally we have also toned down our general conclusions to make sure that we do not overstretch ourselves in making claims, while retaining the striking conclusions that we believe will of wide interest across the field and more broadly across biology.

[Page 6 Line 146]
In the Figure 2 caption the authors' provide rate constants for the reaction at different pHs and temperatures but no detail is provided as to what rate equation they used to determine these rate constants. Are these rate constants experimental first order rate constants?

Yes, these were experimental first-order rate constants using the standard equation $r = \Delta[ATP]/\Delta t$. These were measured over the first 1 hour of reaction to ensure direct comparison between the reactions at 20 °C and 50 °C. So all the rates reported are calculated for the first hour of reaction. This is now included in the paper in the Results section on page 9, lines 246-247.

[Page 7 Line 183]
The authors perform a control experiment whereby the reaction was performed in the absence of Fe^{3+} catalyst (Figure 2d). No ATP was detected in this control experiment which demonstrates that the formation of ATP requires Fe^{3+}.

However, in order to prove the reaction requires acetyl phosphate an additional control experiment is required whereby the reaction is performed in the presence of Fe^{3+} but no acetyl phosphate. The reason this control experiment is required is because it is also possible that ATP could be produced from the reaction between two ADP molecules, i.e. ADP + ADP -> ATP + AMP, and this reaction could be catalysed by Fe^{3+}. This reaction is known in the literature, see W. Huck Nat. Commun. 2021, 12, 5517.

As noted above this control experiment had been done but not included previously (our omission) so it is now included as SI Fig. 1. It confirms that Fe^{3+} did not catalyse the disproportionation of ADP to form AMP + ATP.

It is notable that in the HPLC chromatogram in Figure 1b that a significant peak of AMP is also present. This could indicate that the ATP (at least partially) formed via a 'disproportionation' reaction between two ADP molecules. The authors need to perform this control experiment to confirm whether or not acetyl phosphate is fully or only partially responsible for the phosphorylation of ADP.

The AMP peak is in fact much smaller than the ATP peak, and we imagine simply reflects the hydrolysis of ADP (or ATP formed in the reactions described). The additional control added as SI Fig. 1 should allay any lingering doubts on this point.

[Page 8 Line 197] and [Page 14 Line 426]
The authors demonstrate that, in addition to acetyl phosphate, carbamoyl phosphate (CP) can also phosphorylate ADP to ATP in the presence of $Fe3+$ albeit in a lower yield than for AcP. This is a very nice result as it demonstrates that there are multiple prebiotic routes to the formation of ATP with $Fe3+$.

The authors correctly attribute the lower yields of ATP with CP to the decomposition of CP into Pi and cyanate. The citation of Jones and Lipmann (Ref 57 and Ref 74) is well received as they demonstrated that carbamoyl phosphate is formed in equilibrium with cyanate and Pi.

Given this good result with carbamoyl phosphate I am genuinely surprised at how dismissive the authors are of the importance of cyanate in prebiotic chemistry when it comes to the Discussion. The easiest prebiotically plausible route to carbamoyl phosphate is through a reaction between cyanate and Pi. While I appreciate that in extant metabolism carbamoyl phosphate is formed primarily through a glutamine deamination pathway which releases ammonia which then reacts with carboxy phosphate to form carbamoyl phosphate, this set of reactions is significantly less facile on the prebiotic Earth than the reaction with cyanate.

We accept R2's points here that we had been too dismissive in the previous draft, so have modified this section and incorporated the two passages in the previous MS into a single paragraph on page 13. We have also added some new material on pages 13-14, lines 393-400, on other phosphorylating agents including DAP (as requested by R4). However we stress that our paper is not intended as a review of prebiotic phosphorylation, which is not a small literature, so we have kept these remarks brief.

Furthermore the authors state [Page 15 Line 431]: 'That was important as it showed that biologically relevant condensations are possible in water, but differed from modern biochemistry in that cyanate does not feature in extant metabolism.' This is not correct. Cyanate does feature in extant metabolism. As already mentioned above, and indeed cited by the authors, Jones and Lipmann (Ref 57 and Ref 74) demonstrated that in solution carbamoyl phosphate is in equilibrium with cyanate and Pi and therefore cyanate will be present in cells.

The offending passage has been deleted. Nonetheless we do not really share R2's enthusiasm for carbamoyl phosphate or cyanate as it is far less of a central fit to metabolism than acetyl phosphate. We have therefore been careful not to dismiss its potential relevance and also refer to CP as a 'living fossil' alongside AcP; but we also qualify that it tends to prefer primary N-carbamoylation reactions in modern cells (for example in pyrimidine synthesis).

Ultimately, I believe that this dismissal of cyanate weakens the paper and undermines the authors' own promising result that $Fe3+$ catalyses the phosphorylation of ADP by carbamoyl phosphate. I consider that the demonstration of multiple pathways to the formation of ATP to be a strength of the paper and not a weakness.

We hope that we have addressed R2's concerns above. We no longer dismiss CP. We have also added in a passage about how or why ATP might have displaced AcP and CP as prebiotic phosphoryl donors in the context of a developing protometabolism at the outset of polymerization. This new section is on page 15 lines 451-464.

One experiment the authors could try to raise the yield of ATP with carbamoyl phosphate is to perform the reaction in the presence of excess cyanate which will shift the equilibrium between CP and cyanate + Pi towards carbamoyl phosphate formation. Such a strategy was used in recent publication whereby excess cyanate enabled carbamoyl phosphate formation which in turn facilitated the formation of a phosphoramidate imidazole phosphate that was also demonstrated to phosphorylate ADP to ATP (W. Huck Nat. Commun. 2021, 12, 5517). A possible downside to the addition of excess cyanate here could be that coordination of cyanate to the Fe3+ could interfere with its ability to act as a catalyst. Nevertheless, I think it would be interesting reaction to try.

We have cited the Huck paper mentioned here in relation to this point but hope that R2 will appreciate that these ideas take us a long way away from the focus of this paper on the surprising phosphorylation of ADP to ATP by AcP in water, which resembles the chemistry of life. In any case, in following this course of experiments we would potentially be interfering with the Huck group's own work.

Another interesting set of reactions to try would be whether the Fe3+ catalysed CP phosphorylation also works with the other NDPs or whether it only works with ADP.

We agree that this would be interesting, but as noted above this is really the subject of another paper.

[Page 9, Line 230]
One strand of evidence that the authors claim for importance of the nucleoside moiety in the formation of ATP is that they perform an experiment with pyrophosphate and do not observe triphosphate formation. They analyse this reaction via 31P NMR spectroscopy and the results are shown in Figure 4f and SI Figure 4. The authors note that this analysis is complicated by the paramagnetism of Fe3+ which can reduce signals in the 31P NMR spectra but nevertheless describe this effect as 'cosmetic and does not conceal the absence of triphosphate in the reaction mixture'.

I have significant concerns with Figure 4f and SI Figure 4. In particular, I am concerned with the result for the reaction of acetyl phosphate with pyrophosphate in the presence of Fe3+ as from the 31P NMR spectra (SI Figure 4, green and purple traces) as no inorganic phosphate Pi is present after 5 h. I find it inconceivable that acetyl phosphate was not hydrolysed during this reaction as acetyl phosphate is shown to hydrolyse significantly in the absence of Fe3+ (SI Figure 4, orange and yellow traces). I am very sceptical that Fe3+ inhibited the hydrolysis reaction - if anything I would expect it to catalyse the hydrolysis. Given the glaring absence of Pi here, I do not accept the authors' description that the effect of Fe3+ is 'cosmetic'. If, for example, the Pi was lost during the solid-phase extractions then could this not also have happened to any triphosphate formed during the reaction? In summary, SI Figure 4 does not substantiate the authors claim that the adenosine
is necessary to produce phosphorylation of a diphosphate.

We reluctantly agree with R2 here and have decided to eliminate the NMR work from this paper, as the data presented were the best that we had, and were themselves hard fought. We do not believe the paper loses much if anything for them being cut.

I believe that the authors are correct in their assertion that Fe^{3+} does coordinate to the adenosine as it is well evidenced in the literature that metal ions coordinate to the adenosine nucleobase (H. Sigel Chem. Soc. Rev., 1993, 22, 255-267) but that the experiment performed here is inadequate to prove this.

Instead I would suggest that the authors consider using EPR spectroscopy measurements on the NDP-Fe^{3+} complexes to gain mechanistic insight into their very interesting results. EPR spectroscopy can be performed upon solutions and in conjunction with electronic structure modelling studies can be used to give mechanistic detail. As the authors are in the U.K. I would suggest contacting at Professor Victor Chechik at the University of York who I know has extensive experience in EPR spectroscopy and mechanistic studies.

We are relieved that the Academic Editor does not insist on this EPR work. While we agree that it would be potentially valuable (and we will attempt to set this up in due course) it would take many months to develop this experimental work. Instead we have performed a series of molecular dynamic simulations that give excellent mechanistic insights, as recommended by R1 and R4. We do believe that this greatly strengthens the paper despite the lack of EPR work.

[Page 9 Line 243] and [Page 10 Line 273] and [Page 11 Line 306]
The authors attribute only observing phosphorylation of ADP and not upon other NDPs to the coordination of the N-6 on adenosine to Fe^{3+} while other nucleobases lack an N-6 amine and therefore are unable to coordinate to Fe^{3+}. This leads the authors to propose the formation of macro chelate complex between the N-6 on adenosine, Fe^{3+} and the pyrophosphate (Figure 6).

I strongly disagree with the coordination between the N-6 and Fe^{3+} and therefore I believe their proposed macro chelate complex structure in Figure 6 is incorrect. There is little to no evidence in the literature that the N-6 position of adenosine participates in the formation of a macro chelate complex that the authors describe

Coordination chemistry dictates that the N-6 amine on adenosine should be the second least able nitrogen on adenosine to coordinate to Fe^{3+} (after the N-9 which is bonded to the ribose). This is because the lone pair on the N-6 position can participate in the aromatic system on the adenosine ring and is therefore poorly available to coordinate. The lone pairs on N-1, N-3 and N-7 are orthogonal to the aromatic ring and therefore can effectively coordinate to metal centres.

We agree with R2's points here and have modified the paper accordingly. We are pleased that our new MD simulations also confirm the suggestions made by R2 in this section. As noted above, we have now incorporated two new figures on MD data (Fig. 6 and SI Fig. 7), revised the figure showing our proposed mechanism accordingly (Fig. 7) and added substantive new text in the Results on page 10 lines 269-288 and in the Discussion on page 12, lines 330 to 337.

Numerous studies have shown that in solution a macro chelate complex is formed between N-7 on adenosine, a metal ion and the pyrophosphate and not the N-6 position. See H. Sigel Chem. Soc. Rev., 1993, 22, 255-267 and references therein for an overview of this topic. For specific evidence of ADP-N-7 macro chelate complex with a metal ion, see H. Sigel J. Am. Chem. Soc. 1983, 105, 5891-5900. This paper also shows that a lower percentage of the M^{2+} ADP macro chelate complex is in the closed form than for GDP and IDP complexes (see Table V).

We thank R2 for pointing us to these papers by Sigel, which we now duly cite.

The authors dismiss the importance of the N-7 macro chelate complex to the phosphorylation chemistry stating [Page 9 Line 247]: 'We infer that the critical moiety in the adenosine ring for phosphorylation by AcP with Fe^{3+} as catalyst must be the N6-amino group of adenosine, as the IDP and GDP ring structures are equivalent elsewhere. In particular, from a mechanistic point of view, we note that the N7 is equivalent in all three purine rings, so although this might also interact with Fe^{3+}, as suggested by others [60-63], it cannot be the critical moiety.'

However, the N-7 position on all three purine rings of adenosine, inosine and guanosine are not equivalent from a mechanistic point of view. In their thinking the authors have neglected to consider that replacing the amine with a carbonyl will affect the properties of other N's in the purine ring. Sigel has demonstrated that the basicity of the N-7 position varies significantly as the pKa of the N7-H+ conjugate acid is pKa (Adenosine) = - 0.2, pKa(Inosine) = 1.06 and pKa(Guanosine) = 2.11 (H. Sigel J. Am. Chem. Soc. 1994, 116, 7, 2958-2971). [While this study focused on NMPs I would expect a similar trend in the basicity of the N-7 position for NDPs and NTPs.] Note that this is a log scale so there is a 100-fold difference in the basicity between adenosine and guanosine. The pKa of the N-7 position has a significant effect on the formation of the macro chelate complex with higher basicities leading to higher proportions of the metal ion nucleotide complex being in the macro chelate form (see Figure 4 in H. Sigel J. Am. Chem. Soc. 1994, 116, 7, 2958-2971).

The differences in basicity between the N-7 positions on the purine nucleobases will affect the strength of the coordinate bond (and possibly the extent of backbonding between the metal centre and the aromatic system on the purine) which will therefore affect both the energies and spatial size of the orbitals on the Fe^{3+}. The ability of Fe^{3+} ADP macro chelate complex to catalyse the phosphorylation of ADP will depend on its ability to stabilise the transition state. I would suggest to the authors that the reason they only observe phosphorylation on ADP and not GDP or IDP is because of these aforementioned differences in the Fe^{3+} orbitals that happen to be just right to stabilise the phosphorylation transition state with acetyl phosphate.

Again we thank R2 for these extremely helpful comments. We have referred to the Sigel papers cited here, noted the correspondence to our own MD simulations on page 12 lines 334-337, and touched on macro-chelate complexes and stabilized phosphorylation transition states briefly on page 12, lines 354-355.

The lack of phosphorylation on CDP and UDP here can be attributed to CDP and UDP (as well as their NMPs and NTPs) not forming macro chelate complexes with metal ions and the only interaction in these cases is between the phosphate and the metal ion (see Sigel Chem. Soc. Rev., 1993, 22, 255-267). This is explained in part by the nucleobase more favourably adopting an anti conformation (as opposed to the syn conformation) in relation to the ribose which results in the N-3 facing away from the phosphate/pyrophosphate/triphosphate.

We agree with this and have mentioned that on page 12 line 341.

Later on in the Discussion [Page 11 Line 369], the authors cite a Mossbauer spectroscopy study for evidence of N-6 binding of Fe^{3+} to ADP (Ref 60 I. Rabinowitz J Am Chem Soc. 1966, 88, 4346-4354). I do not believe that this study gives adequate evidence for N-6 binding. First, this study was conducted in 1966 and all later studies contradicted the finding of N-6 binding and have shown that the binding is instead to N-7. Secondly, Rabinowitz et al. make the same mistake as the authors in comparing the results from inosine to adenosine to justify the choice of the N-6 position without considering how replacing the amine at the 6-position with a carbonyl will affect the basicity of other N in the purine nucleobase. Thirdly, Mossbauer spectroscopy is conducted on solid samples (in the Rabinowitz paper the pH of the sample was adjusted and then lyophilised to a powder). The change from solution to solid state means that the results are less reliable than the techniques in later studies which analysed the structure of the metal ion ADP complexes in solution.

In summary, I believe the weight of evidence is that an ADP N-7 macro chelate complex is formed in solution and not the N-6 macro chelate complex and consequently the authors need to adjust their text and Figure 6 to reflect this.

We agree with R2 that our original mechanism was incorrect as regards the N6 amine group and the N7, and we have corrected the paper accordingly, including with the substantial set of new MD simulations discussed above. We trust that R2 will be much more satisfied with the revised mechanism presented in Fig. 7 and justified by the MD simulations detailed above.

[Page 9 Line 257]
The authors show that the addition of low Mg^{2+} and Ca^{2+} concentrations to the reaction leads to higher yields of ATP. The authors justify this increased yield by stating that the Mg^{2+} and Ca^{2+} can stabilise the ATP to hydrolysis which is the explanation also provided by Kitani for this phenomenon. However, the authors go further and claim that the addition of Mg^{2+} and Ca^{2+} promote catalyst turnover by liberating the Fe^{3+}. This would suggest that the rate determining step for the reaction is the liberation of the Fe^{3+}. I am sceptical of this claim as I would have thought the phosphate transfer from AcP to ADP would be the more likely rate determining step. I would favour the explanation that the Mg^{2+} and Ca^{2+} stabilise ATP to hydrolysis. If the authors wish to prove that Mg^{2+} and Ca^{2+} are present in the rate determining step in the catalytic cycle then they would need to demonstrate that the reaction order is dependent on these ions.

We have modified the text here to emphasise the likelihood that Mg^{2+} and Ca^{2+} do in fact stabilize ATP against hydrolysis, though we do not present any specific evidence for this here. We have retained the other possibility that Mg^{2+} and Ca^{2+} might displace Fe^{3+}, but couched it in speculative terms, to make clear that we are only presenting this idea as a possible explanation that requires further work. In fact, the effect of Ca^{2+} and Mg^{2+} is relatively small in any case so we do not really feel that it is deserving of the extended studies that would be required to demonstrate a rate-determining step. Nonetheless, to protect ATP from hydrolysis one suspects that Mg^{2+} would need to interact with the beta and gamma phosphates of ATP, which de facto would require the loss of Fe^{3+} from this position, as is illustrated in Fig. 7. We make it clear in the revised legend that this mechanistic aspect is uncertain.

An alternative explanation for the dependence on Mg^{2+} and Ca^{2+} is that they participate in the formation of a dimeric complex (e.g. FeMg[ADP2]) which is also catalytically active.

We are not persuaded that ADP stacked dimers facilitate phosphorylation by AcP; this goes against our own MALDI-ToF data, plus even more strikingly, the MD data shown in Fig. 6b. We return to this below where R2 raises the point more forcefully.

[Page 10 Line 261]
The authors make an assessment of whether Fe^{3+} is acting as a catalyst by measuring the rate of the reaction with different concentrations of the ADP substrate and then apply a Michalis Menten fitting to the data (Figure 5b). In these experiments the authors use [Fe^{3+}] = 0.5 mM and they don't state the concentration of AcP but I presume it is 4 mM. The ADP concentration is then varied between approx. 0.1 mM - 4 mM. In these circumstances the application of a Michalis Menten kinetic analysis is technically speaking not appropriate as the Michalis Menten kinetic analysis assumes that the substrate concentration is in significant excess over the catalyst concentration (a 10-fold excess of substrate is usually regarded as the minimum viable). This is because the Michalis Menten kinetic analysis assumes pre-equilibrium formation of the enzyme substrate complex and rate determining kcat. In the experiments performed by the authors the concentration of ADP is either lower than Fe^{3+} or at most in an 8-fold excess. Thus, this Michalis Menten fitting is strictly speaking not valid although I do accept that the level off is indicative of catalysis.

We accept that our curve is not strictly a Michaelis-Menten analysis, and we do make it clear that it only resembles a Michaelis-Menten plot. We are pleased that R2 agrees that the level-off indicates catalysis, which was really our main point; we had not intended to imply that a metal ion should conform strictly to enzyme kinetics.

Like for Figure 1, insufficient detail is provided by the authors as to how they measured the rates of reaction here. I appreciate the effort the authors have gone to in order to measure the rate of reaction and this makes a good addition to the study but I would like to see the kinetic data and equation used to determine the rate constant for the data in Figure 5b put into the Supplementary Information so that I can properly assess this aspect of the study.

This is now explained briefly in the Results section on page 9, lines 246-248.

[Page 10 Line 267] and [Page 11 Line 313]
The authors claim that a dimeric structure of ADP with Fe^{3+} (SI Figure 6) is not the catalytically competent species based upon a MALDI-TOF analysis which did not show the stacked species.
I would dismiss the validity of using MALDI-TOF to assess this claim on two criteria. First, the MALDI-TOF is performed on a solid sample in a matrix and not upon a solution and thus the species present may be altered by the change in state. Second, the MALDI analysis requires sample preparation steps that could have significantly interfered with this stacked dimer species. The main drive force behind why the stacking occurs in solution will be to minimise the hydrophobic surface area presented to the solvent by the nucleobases. During the MALDI sample prep the sample is washed with a 50:50 acetonitrile:water solution and acetonitrile is a significantly non-polar solvent it could disrupt the hydrophobic stacking and thereby break this stacked dimer apart.

I think the authors should remove this MALDI study from the paper as it is not possible that this experiment can give any effective insight into whether a dimer is formed during the reaction in solution.

We do not agree with R2's points here. We have quite a lot of experience of using MALDI-ToF to measure stacking – experience that we regretted acquiring as we had initially detected stacks while analysing AMP solutions for evidence of polymerization in the presence of AcP. We were initially excited about the possibility of polymerization, but later realised that it was simply stacking. In fact we detected stacks of up to 7 nucleotides in length from AMP (and have also explored stacking using MD simulations). In this study we used the same instrument and the same methodology but ADP in place of AMP. In this case, we were unable to detect any stacking at all. Nonetheless, in deference to R2's expertise, we have been careful to frame these results with circumspection. We would certainly not dismiss dimers were it not for the fact that the MD simulations make it surprisingly clear that interactions between two ADPs actually severely limit the proportion of simulation time in which the ADP beta phosphate is positioned close to the AcP phosphate. We therefore believe that dimers genuinely do not play an important role in the phosphorylation reactions observed here.

Furthermore, the evidence from the literature that metal complexes can form dimeric structures (with intermolecular adenosine N-7 metal ion interactions) and can also stack is fairly substantial, see H. Sigel J. Am. Chem. Soc. 1983, 105, 5891-5900. I would therefore not dismiss the possibility that the catalytically competent species for the phosphorylation reaction is the dimeric form. Note too that the optimal 1: 1 stoichiometry for the reaction applies to both the monomeric complex (1:1) and the dimeric complex (2:2) and therefore cannot be used to distinguish between these mechanisms.

We appreciate the point about stoichiometry and dimeric complexes but feel that our points above merit our argument that stacking is unlikely to play a role in this case. We feel that the combination of MALDI-ToF and MD simulations can and do distinguish between these two possibilities and strongly suggest (if not unequivocally) that the mechanism given in Fig. 7, involving monomers only, is the best approximation to the truth from our data.

[Page 11 Line 301]
As acknowledged by the authors, there is not a link between Fe3+ and the phosphorylation of ADP to ATP in biology. I would agree with this as to the best of my knowledge ATP synthesising enzymes do not make use of Fe3+ as a cofactor. For example, the acetate kinases in Ref 41 use Mg2+ as a cofactor. The authors advocate a position on the origins of life whereby the chemistry out of which life emerges should align as closely as possible to the biochemistry of extant life. If Fe3+ were to play such a crucial role at the origins of life then why would this not have been retained in biology? Such a question is raised by the authors but they do not provide any adequate answer to it.

We have now answered this question explicitly on page 12, lines 356-362. We would imagine that the combination of limited availability (relative to the high requirement for ATP synthesis) combined with potentially dangerous reactivity (such as Fenton chemistry) would have provided a strong selection pressure for enzymatic catalysis to displace any ancestral role of Fe3+.

[Page 11 Line 296] and [Page 15 Line 447]

At the opening of the Discussion the authors state that: 'Taken together, these findings suggest that the pre-eminence of ATP in biology has its roots in aqueous prebiotic chemistry. … This implies that ATP

became the universal energy currency of life not as the endpoint of genetic selection or some frozen accident, but for fundamental chemical reasons, and probably in a monomer world before the polymerization of RNA, DNA and proteins.' And then end the Discussion stating: 'If so, then ATP became established as the universal energy currency for reasons of prebiotic chemistry, in a monomer world before the emergence of genetically encoded macromolecular engines.'

The authors claim that their results justify why ATP became the universal energy currency for life and moreover did so before the advent of enzyme catalysts. This is a bold claim and unfortunately the authors have significantly overextend themselves in making it as such a claim cannot be fully supported by their results.

We have added a few words to tone down these claims, as we agree they are not fully supported by the results, though we think they are perhaps better supported than R2 suggests here (see next paragraph).

My main objection here is that the authors have only demonstrated half of what is necessary to solve why ATP became Life's universal energy currency. To justify why ATP became Life's energy universal currency at the origins of Life requires consideration of both how ATP could have formed and how ATP came to be not just the dominant phosphorylating NTP but also the dominant phosphorylating reagent at the origins of Life.

The authors' results do provide a plausible explanation for why the formation of ATP became dominant over the other NTPs but this is as far as the authors can reasonably take their conclusions based upon their results.

The huge oversight by the authors here is that they do not seriously consider how ATP performed prebiotic phosphorylations. This receives scant attention in the paper and is only addressed in the penultimate sentence. The authors state [Page 15 Line 443]: 'Once formed, ATP would promote intermediary metabolism through phosphorylation'.

We now give more context for life as a guide to its own origins, which makes a stronger case based on data from other recent work. This new material is in the Introduction on pages 3-4, lines 64-83 and in the Discussion on page 15 lines 451-464. While we accept that these data are not presented directly in our paper, and are therefore careful to tone down our claims, we believe that the added context makes the basis of the argument much clearer than perhaps it was in the previous version.

This statement trivialises an enormous challenge in prebiotic chemistry which is how to perform prebiotic phosphorylation reactions. For example, a central challenge to prebiotic phosphorylations is how to overcome the high activity of water which results in the hydrolysis of the phosphorylating reagent rather than transfer of phosphate to an organic molecule. The authors show that solutions with a high water activity favour formation of ATP [Page 6 Line 166], however such conditions will be the least effective for the phosphorylation of organic compounds meaning that the authors will almost certainly need a very different set of conditions for the phosphorylation by ATP. Under these different conditions would ATP necessarily be a better phosphorylating reagent than the other NTPs? Furthermore, I would argue that ATP is a very poor choice as a prebiotic phosphorylating reagent. This is because ATP is a very stable compound that has a hydrolysis half-life of around 2 years at pH 8 and 25 oC in synthetic sea water (H. Hullet Nature 1970, 225, 1248). Given the high stability of ATP I would not

expect its use in prebiotic phosphorylations until after the advent of macromolecular engines e.g. a ribozomyl or enzyme catalyst.

This may be so, but we note that at 50 °C we can see ATP hydrolysis taking place right in front of our eyes in Fig. 3d and e. In hydrothermal environments it seems likely that ATP turnover would indeed be rapid. We agree that we have not yet demonstrated which catalysts might give ATP an advantage over other phosphoryl donors, but we do provide two pivotal reasons for the dominance of ATP in the context of life as a guide to its own origins – the requirement for nucleoside triphosphates for polymerization of both RNA and proteins (which is now presented more clearly in paragraph 2 of the Introduction, on page 3, and the autocatalytic requirement for ATP to make ATP during purine synthesis, which is raised on page 4 of the Introduction, lines 78-83. We have also added another paragraph to the Discussion which discusses exactly the points raised by R2 here, on pages 15 and 16, lines 451- 464 – which catalysis and at what stage of evolution would we expect ATP to begin displacing alternative phosphoryl donors. Of course we accept that this remains tentative, but it is clearly testable, and it is in the interests of science that we lay out this appealing hypothesis with clarity to enable others besides ourselves to test it.

If the authors wish to demonstrate why ATP became Life's universal energy currency then they must consider questions such as the following and address these experimentally:
Why did life settle upon NTPs to perform reactions in prebiotic chemistry?

This is explicitly discussed in the Introduction paragraph 2

Why did ATP become dominant phosphorylating reagent over the other NTPs?

Through positive feedback loops, and for the simple reason that it could be formed before enzymes and genes, building it into the core of biochemistry from a very early stage of evolution.

How did phosphorylation of organic compounds by ATP overcome the high activity of water?

This does not seem to be an issue with the phosphorylation of ADP to ATP in water, which ironically works better in pure water than at lower water activity. We presume metal ion catalysts, or possibly organic catalysts.

If, as the authors show, ATP can be selectively formed be a metal ion catalysed reaction could it not also perform metal ion catalysed phosphorylations that other NTPs cannot?

This is certainly possible, indeed probable, but we have no evidence for that at this time.

Given the stability of ATP/NTPs Is it not possible that ATP is chosen 'late' in the origins of Life after the origin of macromolecular engines?

This seems to us extremely doubtful for the reasons given in paragraph 2 of the Introduction – NTPs are absolutely central to all forms of polymerization in cells and there is every reason to think that ATP was available before polymerization and macromolecular engines – evidence presented in this paper.

The experimental results presented in this paper do not address these questions and therefore do not tackle how ATP could perform prebiotic phosphorylations and thus for the authors to claim that their

results show why ATP became Life's universal energy currency is not justified. The authors need to constrain their conclusions to what their experimental results can actually prove.

We believe our results go some of that way, and the context from our own work and that of others makes many of the other points quite forcefully, as noted above. We therefore agree with R2 that we should be careful to couch our claims carefully, but we believe that the results presented here, in the context presented in the revised paper, make a strong case for why ATP became the universal energy currency.

The authors must also consider how prebiotic chemistry also produced the other NTPs as Life has settled on the use of NTPs for synthesis of DNA and RNA. This requires an alternative route(s) to NTPs. If this alternative route(s) to NTPs produced all NTPs at a higher rate than the Fe^{3+} catalysed phosphorylation of ADP by acetyl phosphate then this would undermine the authors' case that ATP was formed in excess over other NTPs.

We agree that this is an unanswered question at present and will be looking into this in future work.

Minor considerations:

In the caption of Figure 4 it would be better to call it a nucleobase rather than base.

OK

For Table 1, the authors may wish to cite a recent paper on the geological presence of cyclic trimetaphosphate: Britvin, S. N. et al. Cyclophosphates, a new class of native phosphorus compounds, and some insights into prebiotic phosphorylation on early Earth. Geology 49, 382-386 (2020)
https://eur01.safelinks.protection.outlook.com/?url=https%3A%2F%2Fdoi.org%2F10.1130%2FG48203.1&data=04%7C01%7Cnick.lane%40ucl.ac.uk%7C5c56192ff2454d830dea08d9be480628%7C1faf88fea9984c5b93c9210a11d9a5c2%7C0%7C0%7C637750039203076173%7CUnknown%7CTWFpbGZsb3d8eyJWIjoiMC4wLjAwMDAiLCJQIjoiV2luMzIiLCJBTiI6Ik1haWwiLCJXVCI6Mn0%3D%7C3000&sdata=jMB%2BVa5MCZbsfLkhG%2F8Rvl9rok3h0huO%2BAbuXrG4KBU%3D&reserved=0
[Page 8 Line 207] with regards to cyanate being a condensing agent, in addition to ref 58 the authros may wish to also cite R. Pascal J. Am. Chem. Soc. 2006, 128, 7412-7413.
Mathew Pasek wrote a very good recent review of prebiotic phosphate chemistry that the authors may wish to cite: Chem. Rev. 2020, 120, 11, 4690-4706 10.1021/acs.chemrev.9b00492

We thank R2 for these suggestions and have included these references. Once again, we thank R2 for this extremely thorough and helpful review which has made a huge difference to paper. We are extremely grateful.

Reviewer #3:

This is an interesting paper which relates to a chemical, non-enzymatic synthesis of ATP from ADP and Pi using acetyl phosphate and ferric ion. From a chemical perspective alone, the work is not necessarily original but it is more in the context of prebiotic chemistry and abiogenesis that the work is couched and consequently, it has been reviewed here in that context.

We thank R3 for their review and valuable perspective.

It is pertinent to comment first on the authors contextual history in the field which is significant. The authors have a long-standing presence in the sphere of prebiotic chemistry, specifically linked to the role of concentration and charge gradients as a source of energy to drive the emergence of biology.

The present contribution centres on the conversion of adenosine diphosphate (ADP) and inorganic phosphate (Pi) to adenosine triphosphate (ATP) using acetyl phosphate and ferric ion as co-factors, without the need to invoke multi-subunit protein complexes as catalysts. There a number of intriguing aspects to this work, including (i) the specificity of acetyl phosphate in this process (although carbamoyl phosphate also shows some activity) and (ii) the specificity of ferric ion. Both of these are sufficiently intriguing as to ask why these components appear to be so unique and what is the level of significance in this from a pre-biotic perspective.

We thank R3 for this concise and generous synopsis.

From a practical methods perspective, these looks perfectly sound to me, chemically and analytically. Technically, relatively little discussion is devoted to the kinetic aspects of the study but the focus is more on thermodynamics. From a prebiotic energetic context this is certainly of value, however, within the context of dynamic kinetic behaviour as in biology, it tells only a part of the story.

We are not quite sure which aspects of the kinetics R3 would like us to address in more detail, but note that we have added in extensive discussions of mechanism based on molecular dynamic simulations, which we hope will address some of these concerns. Also see the next paragraph below.

In terms of comments, I have two to pass on to the authors for their consideration: (i) it is clear that the authors place great emphasis on the specificity of both acetyl phosphate and ferric ions in this chemistry, it is central to the actual conclusions. Figure 6 proposes a potential mechanism for how this might work. However, what is rather surprising missing is a more thorough molecular modelling analysis of just how reasonable this actually is. This is a significant omission and one that could logically be quite straight-forward to add. I would not suggest full molecular dynamics calculations be necessary at his stage but simple bench-top molecular mechanics modelling should be included.

We thank R3 for this suggestion, which we have taken, and we agree that it has made a major difference to our paper, as well as altering the exact mechanism proposed. We have laid this out in response to the other reviewers, but specifically, we have added two new figures on MD data (Fig 6 and SI Fig. 7), revised the figure showing our proposed mechanism accordingly (Fig. 7) and added substantive new text in the Results on page 10 lines 269-288 and in the Discussion on page 12, lines 330 to 337. We agree that these MD simulations substantially improve the paper.

(ii) The second point is rather more contextual, but goes to the central importance of the present chemistry in primitive energy transduction. The authors emphasise the value of ATP as a primitive energetic "battery". This has some validity of course, but I feel it really does need to
be nuanced a little more for the wider and especially biological audience. For sure, ATP can act as an energetic battery in terms of direct phosphorylation. However, the situation becomes more complex

when it comes to linking ATP/ADP to proton gradient or indeed to enzymatic phosphorylation-dephosphorylation. The energy which results from ATP hydrolysis in biological systems is transduced via multi-subunit complex enzymes and converted to mechanical energy through a rotatory mechanism. Because the actual hydrolysis reaction of ATP is enzymatically very fast (femtosecond), it is generally believed that this chemical hydrolysis step itself is NOT the step in the whole process where energy is transduced, but rather the binding-de-binding of ADP/ATP components from the enzyme binding sites themselves. This is what allows the ATP hydrolysis to be connected to proton gradients. Non of this is made clear in the current analysis which makes it a little less clear just how significant pre-enzymatic energy transduction using ATP might have been and what could have acted as a pre-enzymatic catalyst and then also, how was that energy transduced from ATP to a gradient or other usable energy source. With a little more nuance, the authors might be able to place their work in a slightly more realistic context within the pre-biotic milieu.

It seems that our previous MS had lost all the reviewers on this specific aspect, so we have revised (and actually shortened this passage to make it more clear). Specifically, we explain that we envisage these processes all taking place within protocells, so the ATP is formed immediately adjacent to the membrane within protocells under the influence of proton influx through fatty-acid flip flop. We anticipate that ATP would drive the polymerization of nucleotides and amino acids in water, in the presence of metal ions or other monomeric (or small oligomeric) organic catalysts. This is outlined in two new sections in the Discussion, on page 15 lines 436-443 and lines 451-464. We have also provided much better context for the idea of life as a guide to its own origin in terms of the origins of protometabolism. This new material is included in the Introduction on pages 3-4 lines 64 to 83. We trust that these new passages will provide the context that R3 was missing in the previous version of the paper.

Once again, we thank R3 for their very helpful comments, and especially the recommendation to perform molecular dynamic simulations, which has made a big difference to the paper.

Reviewer #4:

In "A prebiotic basis for ATP as the universal energy currency" the authors present an intriguing argument that the origin of ATP as the dominant energy carrier for prebiotic chemistry resides in the fact that acetylphosphate can phosphorylate ADP to form ATP (and ADP alone!) in the presence of catalytic Fe3+. Indeed, the authors demonstrate that all three of these components are uniquely situated to perform this task: changing any nucleobase, metal catalyst, or phosphorylating agent and the yields plummet. Given the specificity of adenosine in this task, the authors argue that ATP was predisposed to be the dominant energy currency of life.

In general, the experiments are well-thought out and easy to follow. The analytical tools appear reasonable, within the limitations of these instruments. The reasoning within the discussion is sound enough (in general) for the experiments that have been carried out.

We thank R4 for these positive words.

I have several concerns regarding the conclusion, other phosphorylation agents that were not discussed in the text, the prebiotic geochemistry of Fe3+, and some of the methodology and reporting of results. These are explored below.

1. The main point of the paper is that ADP to ATP occurs readily only by phosphorylation by acetylphosphate with catalytic Fe^{3+}, under a specific set of aqueous conditions (acidic pH, T <= 50°C, lacking too much salt or divalent cations). Thereby, the conclusion is that ATP as the dominant is not a "frozen accident" but is a consequence of the environment in which life originated. However, such a conclusion appears to be a circular argument: the specificity of conditions needed to generate ATP from ADP means that ATP was specifically chosen because of those conditions. If the conditions are not met on the prebiotic earth (e.g., no Fe^{3+}, no acetylphosphate), then no ATP would be specific. There are other routes to generating prebiotic NTPs (detailed in points 2+3). If ATP is a "frozen accident" then these routes would be just as plausible. To some extent, this point is allayed by the discussion on acetylphosphate, as acetylphosphate is present in modern biology.

This is an interesting point, and we agree that it is somewhat allayed by the context of life as a guide, which we have emphasised in the revised MS.

However, if we presume the earth was somehow "different" with respect to its prebiotic geochemistry (see Benner et al. ChemSystemsChem 2020), and thereby other reactants not observed in geochemistry or biochemistry today would have been plausible (e.g., cyanate as discussed by the authors), then prebiotic chemistry need not follow modern biochemistry.

This is true, but makes an assumption that we would not necessarily agree with, which is that life could begin in almost any environment. We do not believe this to be true on the grounds that life as we know it is cellular, uses CO_2 and H_2 as the basis for an autotrophic metabolism, and is obligately chemiosmotic. If these traits are not just chance, but have a basis for their existence (and all of them arguably do) then not every environment could support the origin of life. A primary paper is not really the place for this kind of discussion, but we agree that our perspective only makes sense if given better orientation. We have therefore reorganized the Introduction to make a clearer case for life as a guide to the emergence of metabolism. We have made it clear that this is a hypothesis, and duly testable, but that it is only one perspective on the origin of life. Nonetheless we do think it has been an overlooked perspective and is supported by the data presented here.

Prebiotic chemistry is often OK with "frozen accidents" as well, as the chirality of biomolecules is generally assumed to be a consequence of the possibly arbitrary higher abundance of molecules with the biological handedness. Also, some aspects of the genetic code (while highly evolved) were likely randomly frozen in as well.

We have a different perspective on the code, which emerges from the structure of autotrophic metabolism. This will be published shortly in BBA Bioenergetics, and suggests that the code is anything but a frozen accident. But in any case, we have toned down our comments on frozen accidents and tried to frame the argument from the point of view of the abundance and antiquity of adenine nucleotide cofactors in the revised Introduction.

2. The paper is lacking discussion of the amidophosphates, which have been explore by the Krishnamurthy lab for their ability to phosphorylate organics. Gibard et al. (Nat Chem 2018) demonstrated phosphorylation of NDPs to NTPs using diamidophosphate. Recently, Lin et al. (Ange.

Chem 2021, very recently published, but building on Gibard et al. 2018) demonstrated NMPs are transformed into NTPs via reaction with diamidophosphate. Amidophosphates have a parallel in modern biochemistry in forms such as creatine phosphate.

We have added some new material on diamidophosphates and creatine phosphate on pages 13 and 14, lines 393-400. We now cite the papers mentioned by R3. However, despite their excellence in their own right, we do not feel that these papers are strongly relevant to the data and argument developed here, as creatine phosphate is entirely restricted to eukaroytes. To our knowledge no N-P bonds exist in central metabolism common to archaea and bacteria. Phosphoamidates therefore have little relevance to extant biology. We also note that DAP in solution will self-condense to form cyclic trimetaphosphate, as shown in the SI of Gibard et al in a number of figures there. In fact we did consider cTMP in our paper, but it had only limited utility at lower temperatures and alkaline pH, as shown in Fig. 3. This was not an aspect that we wanted to dwell on in the context of our paper.

3. Triphosphorylation of nucleosides via cyclic trimetaphosphate and a ribozyme has been reported by Akoopie et al. (Sci Adv 2021) and presents an alternative explanation for NTPs: post-RNA world (Moretti and Muller 2014). Although trimetaphosphate has its own issues with prebiotic provenance (e.g., Keefe and Miller 1995), some routes have been shown to these compounds (Yamagata et al. Nature 1991, Pasek et al. ACIE 2008). Kim and Benner (Astrobiology 2021) also showed NTPs from trimetaphosphate.

We have been more careful to stress that the context we are framing in our paper is pre-RNA world, indeed before any sort of polymerization. We therefore do not feel that an extended discussion of work on phosphorylation with ribozymes is especially relevant to the paper as written; this is not a review of prebiotic phosphorylation, but specifically about the provenance of ATP in protometabolism.

4. The prebiotic geochemistry and aqueous chemistry of Fe^{3+} is not adequately considered. Fe^{3+} is generally soluble only at low pH, well below the pH used by the authors. Iron redox diagrams (for instance, see Takeno 2005, Atlas of Eh-pH diagrams, Figure 47) show that soluble Fe^{3+} exists only at pH <2.3. Above a pH 2.3, Fe^{3+} reacts with water to produce $Fe(OH)^{2+}$, and then $Fe(OH)_2^{+}$. These are not Fe^{3+}. This chemistry is roughly reflected in the results presented by the authors. At the higher pH, Fe^{3+} precipitates out as $Fe(OH)_3$ or FeOOH. The drop in yield at these higher pH values likely corresponds to a precipitation of Fe^{3+} or the production of neutral Fe species (such as $Fe(OH)_3$ and FeOOH). The actual aqueous ionic chemistry of Fe^{3+} at the conditions being explored (Figure 6) is likely completely different than those being discussed here as Fe^{3+} (aq) isn't Fe^{3+} at a pH of 5.5.

We have added a little more on the availability of Fe^{3+}, but had already included some material in the previous version. In particular, we mention photooxidation in terrestrial geothermal systems, and mixing of hydrothermal fluids and ocean waters in submarine systems. Notably, according to Shibuya et al GCA 2016 (Fig 3), mixing of ocean water and hydrothermal fluids should generate Fe^{3+} in solution between ~30C and ~80C as minerals form slowly and are metastable. We therefore think it is likely that at least some Fe^{3+} would be available in submarine hydrothermal systems as well as terrestrial geothermal systems.

We agree with R3 that Fe^{3+} readily precipitates at alkaline pH and probably even neutral pH. We do note that this is almost certainly why yields were lower at alkaline pH. Nonetheless, the idea that Fe^{3+} is not available as an ion is not borne out by our data. We have included an additional SI figure to show that

chelating Fe^{3+} with EDTA prevents ADP phosphorylation by AcP; small clusters such as 4Fe4S clusters did not work either, nor did surfaces such as hematite. We therefore believe our data support the specific involvement of the Fe^{3+} ion with its high charge density and specific d orbital electronic configuration.

To this end, it's also unclear if the pH of the solution was adjusted before or after the addition of Fe^{3+} (as $Fe_2(SO_4)_3$). I presume after, but if before, Fe^{3+} is an acidic cation that will drive the pH to a lower value.

We adjusted the pH after addition of Fe^{3+} and now state this clearly.

Furthermore, Fe^{3+} requires highly oxidizing conditions at low pH, well above the point of the H_2S/SO_4^{2-} redox couple (a full 0.6 V at pH 2), and given that thioesters are discussed in the context of this prebiotic chemistry, these highly disparate redox conditions themselves would introduce disequilibrium. The arguments for Fe^{3+} could also be expanded in the text, as Fe^{3+} also forms from the reaction of Fe^{2+} with H_2O_2, the latter formed by the photolysis of water (via Fenton chemistry).

As noted above, we have expanded the Discussion of availability of Fe^{3+} a little in the text, but there is a great deal of uncertainty about conditions in the Hadean. This is not a review paper, so we have tried to keep this discussion within a reasonable limit.

In addition, Fe^{3+} is unstable in non-acidic water, eventually precipitating out as hematite (Fe_2O_3). The reaction $2Fe^{3+} + 3H_2O = Fe_2O_3 + 6H^+$ has a reaction K of around 0.7 at 30°C, which means that at a pH of 5.5., the concentration of Fe^{3+} should be sparingly small at equilibrium. That's not to say solutions can't be supersaturated with respect to Fe^{3+}, but that high concentrations of Fe^{3+} should be geologically ephemeral.

We agree with R4 about this. But please note our comments above. In continuous flow environments, we would expect continuous mixing, hence although Fe^{3+} would be precipitating continuously it would also be formed continuously with mixing and oxidation at warmer temperatures (see Shibuya et al GCA 2016 (Fig 3). We agree that conditions are likely to be supersaturated with Fe^{3+} concentrations very low, but it is harder to be more specific than we already are about the possibilities in a Hadean environment.

5. The chemistry of iron (III) is more closely replicated by aluminum (III) than by the other species investigated. The ionic size of Al^{3+} is similar to Fe^{3+} and the chemistry of these ions in water vs. pH is similar. It may be worth replicating these experiments with Al^{3+} to see if Fe^{3+} is still unique in its chemical potential for ADP to ATP, especially in comparison to the species chosen (Co^{3+}, Cr^{3+}, and Mo^{3+} are all quite different from Fe^{3+} in aqueous chemistry).

This is an interesting point, and to be honest it hadn't occurred to us that Al^{3+} might have similar chemistry to Fe^{3+}. We appreciate that it would have a similar charge density, but not being a transition metal, it is hardly comparable in its electronic configuration. In any case, we feel that it would be more appropriate to explore the possible involvement of Al^{3+} in future work rather than this paper, which is already substantial.

6. The results do not discuss the possibility of precipitates of the various components investigated. Does iron(III) form red FeOOH precipitates? Is there any precipitation of Mg-ADP? Is phosphate being precipitated by the cations? This is also a question of mass balance: Is the amount of phosphorus in ADP and AcP that was added to the beginning of the reaction the same as the total organophosphate that came out? If not, were there precipitates of some of these ions (perhaps ATP in some of the Mg/Ca experiments?)? If no precipitates were noticed, then this should be noted in the text. The Fe3+ should certainly provide precipitates, given the "gumminess" of this ion.

We certainly observed precipitates, and especially (as expected) under the more alkaline conditions. We did not specifically consider mass balance, nor did we weigh the precipitates; we assumed them to be the anticipated ferric hydroxides, apatite, brucite, and so on. Precipitation is clearly an occupational hazard of working across a range of pH and temperatures, but we have generally taken it to be a constraint on what is possible. For example, we have recently published a paper showing that precipitation of apatite (calcium phosphate), by adding acetyl phosphate or simply Pi to the formose reaction, stops the reaction in its tracks and in fact stabilises ribose on the mineral surface (Camprubi et al, Astrobiology, July 2022). The amount of precipitate was only part of the point here; the main point is that the catalyst was lost from the system, terminating the formose reaction, and concentrating the earlier intermediates. We do believe something similar is happening here: precipitation of Fe3+ at neutral to alkaline pH prevents the synthesis of ATP under these conditions, but more importantly, this means that ATP formed under mildly acidic conditions (for example under an influx of protons within protocells) would be pushed out of equilibrium when diffusing into bulk alkaline conditions in the cytosol.

7. The study of the pyrophosphate phosphorylation (figure SI 4) could be improved significantly by changing the solution pH of the NMRs acquired. The spectra are quite broad, as acknowledged by the authors. However, given the multiplicity of the PPPi peak, and its expected low concentration (~20% of ADP was converted in these experiments) would a triphosphate peak even be visible if it was at ~5-10% yield in such conditions? The authors could attempt to precipitate Fe3+ using NaOH or Na2S to sharpen up the spectra and investigate for PPPi. As it is, the current spectra do not provide confidence in either its presence or absence.

We completely agree with R4 that the NMR spectra of PPPi are not sufficiently high quality, so we have dropped them entirely from the paper, as noted earlier. We do not think this is a great loss and we had already spent some considerable time attempting to improve them without success.

Once again, we thank R4 for their time and valuable comments on the MS. We believe that our revisions in response improve the paper considerably, and we hope that R4 agrees.

COMMENTS FROM THE ACADEMIC EDITOR:

The manuscript by Pinna et al. characterizes a previously described reaction (as the authors note) whereby acetyl phosphate phosphorylates ADP to produce ATP, catalysed by Fe3+. However, the authors then go on to demonstrate the specificity of the reaction for ADP over other ribonucleotides. That result on its own makes the work publishable (generally speaking). The manuscript is written eloquently, and the message of the authors is clearly expressed. However, there are serious flaws in the interpretation of the data and in the placement of the data in a broader context. Therefore, in my opinion, the manuscript would need to be substantially revised for consideration.

We are extremely grateful to the Academic Editor for their kind take on this paper and constructive suggestions for improvements. We value too the pragmatism that shines through these comments.

All of the reviewers gave constructive criticisms that should be considered. Reviewer 2 provides a longer, extensive list of criticisms. I do not find the requests unreasonable, as several of the points seek to clarify and/or correct shortcomings. However, I would not push for the EPR analysis to be performed. EPR would be insightful, but that would take much time, and most of the criticisms can be addressed without EPR.

Sincere thanks for sparing us that. We agree. We believe that the MD simulations make a big difference to the paper, though, and make large amends for this omission.

As the reviewers have done a thorough job, I will only briefly comment on a few of the points raised. The complex described in Figure 6 is questionable. If this were to be taken more seriously, then some type of modelling and additional experiments would be needed. As reviewers 1, 2 and 3 noted, and as is well known in coordination chemistry, the coordination of metal ions to the N6 position of adenosine is not reasonable since the electrons of N6 are distributed across the aromatic ring. Even when deprotonated at high pH, this position is a poor ligand.

We agree with this too and have done some additional modelling which supports the revised mechanism (which is in fact quite close to what two of the referees had suggested). We have outlined the changes we have made in some detail above in response to the individual referees.

It is also necessary to run a control demonstrating that ADP + ADP ◊ ATP + AMP is not occurring, particularly since this reaction has been documented before (as reviewer 2 notes).

This had already been done and is now included in the paper as SI Fig. 1.

As reviewers 1 notes, the data are consistent with a “surface pond or lake” rather than a hydrothermal vent. It would be better if the authors let their own data guide them rather than to force interpretations onto previously espoused convictions. I can understand if the authors do not want to advocate for surface pond or lake conditions, but a more fair and balanced interpretation of the data are warranted and would contribute well to the field.

We have revised the Discussion to make this much clearer. We have also restructured the Discussion, so that this passage now comes before the paragraphs on work and disequilibrium, which we suspect had distracted attention from the passages on terrestrial geothermal systems before. We believe that we are now explicit that the chemistry described would work best of all in terrestrial geothermal systems, but that this does not rule out other systems including submarine hydrothermal systems, for reasons that we detail in the revised text. We now consider the ADP/ATP disequilibrium in this context, rather than the other way around.

Regarding more fresh water conditions, on pages 4-5 the manuscript states “But the fact that substrate-level phosphorylation of ADP to ATP can be accomplished by AcP in water says nothing about whether his mechanism actually holds prebiotic relevance.” This is a fair point, but the conditions used by Pinna et al. are not necessarily anymore prebiotically plausible. Basically, extremely low levels of salt are necessary to allow for the reaction to occur. The fact that NaCl and $MgCl_2$ greatly inhibits the reaction is alarming in terms of prebiotic relevance, especially if you are a proponent of hydrothermal vents. The fact that 1-2 mM Mg^{2+} promotes the reaction does not strengthen the arguments. Environmental concentrations of Mg^{2+} are much higher. Even the concentration inside of a cell is much higher. Unless one were to invoke freshwater conditions, the inhibition by Mg^{2+} (and Na^+) suggests that the data are not relevant to prebiotic chemistry, as it is difficult to imagine how such chemistry could have been mediated on the prebiotic earth.

We have added in a new section in the Discussion which addresses these points. NaCl does not strongly inhibit the reaction, but high modern ocean levels of Ca^{2+} and Mg^{2+} most certainly do. However, these ions generally precipitate out in alkaline hydrothermal systems, hence their concentrations are typically less than 5 mM and often less than 1 mM, which is entirely consistent with the work presented here. We have made this clear in the revised discussion.

To help the reader, it would be good to indicate what pressures are typically found in hydrothermal vents. That way, the reader would understand what kind of environments the 80 bar data correspond to.

We added in a note on pressures in submarine hydrothermal systems (typically 100-300 Bar).

As reviewer 4 notes, it is alarming that some highly relevant work was overlooked, including work from the Huck group (Maguire et al. Nat Commun 2021, 12, 5517) and the Krishnamurthy group (Gibard et al. Nat Chem 10, 212–217). These are extremely relevant studies that explore prebiotic phosphorylation. If a fair and balanced perspective were to be taken, then the implications of such work should be taken into consideration.

We have now cited these papers, and included a short discussion of their importance in relation to our own study. But in fact we do not think they are strongly relevant to our approach of using life as a guide to protometabolism, and we explain why; of course we credit the fine work that has been done. We have also revised the Introduction and some of the Discussion to make our own approach (life as a guide) more explicit.

Similarly, the neglect of the potential importance of cyanate further suggests biases. This is even more evident since the dismissal of cyanate is inconsistent with how the authors seem to view prebiotic chemistry, in that things that are not found in biology are not considered. Using the same logic, we should dismiss a role of Fe^{3+} in prebiotic phosphorylation, since that is not found in biology.

This is a fair point. We have eliminated the passages that dismissed cyanate and have added in some thoughts on why Fe^{3+} does not catalyse ATP synthesis in modern biology.

Reviewer 4 raises several points regarding the solubility and availability of Fe^{3+}. Additionally, the photooxidation of Fe^{2+} to Fe^{3+} has been experimentally demonstrated (Bonfio et al. Nat Chem 9, 1229–1234).

We now cite this paper and briefly discuss the photooxidation of ferrous iron in terrestrial geothermal systems.

Finally, as reviewer 1 notes, the speculation that ATP would be synthesized at acidic pH and then enter a protocell with an internal alkaline pH does not appear logical. Nucleotide triphosphates are not permeable across membranes that are thought to be prebiotically plausible. If membranes were stable to high concentrations of Mg^{2+}, then perhaps enough Mg^{2+} would be bound to neutralize the charge, but such concentrations of Mg^{2+} would inhibit phosphorylation of ADP to ATP. It is difficult to imagine a scenario in which the gradients that the authors wish to invoke were established.

I'm afraid this was a misunderstanding, based on a poorly written passage in the first draft of the MS. We do not anticipate ATP crossing membranes, but rather imagine it being formed within protocells, in the immediate vicinity of the membrane, subject to an influx of protons via fatty acid flip flop. We have revised the Discussion to make this clear now.

There is something interesting in the submitted manuscript. The specificity for ADP is new and definitely interesting. The details of the chemistry need to be improved, and a fairer representation of the implications of the work and how that fits into prior studies is needed.

We have added a series of molecular dynamic simulations, which support the revised mechanism along the lines suggested by R2 and R3. We have also rebalanced the text to make our own position more clear while also being as fair as we can to the work of others. This is not a review paper, and it is not the place to review all the work that has been done of different phosphoryl donors in prebiotic chemistry (there has been a tremendous amount). Rather, it presents an unexpected and important finding, which broadly supports the idea that ATP could have arisen early in protometabolism following pathways reminiscent of life's metabolism. We believe this work is important and extremely relevant to biologists in particular, who are often perplexed by questions about the universal energy currency and the origins of metabolism. We believe our paper has important things to say about both of these areas, grounded in beautiful data and with a well-supported mechanism. It also makes the unusual point that phosphorylation reactions can take place in water perfectly well, in the absence of enzymes, and with the simplest metal catalysts. All these points will challenge the field and resonate with biologists. We therefore hope that you will see fit to publish this extensively revised and improved paper in PLOS Biology.
