# Supplementary material for: A prebiotic basis for ATP as the universal energy currency
Source: PLoS Biol. 2022 Oct 4;20(10):e3001437. doi: 10.1371/journal.pbio.3001437 (PMC9531788; doi:10.1371/journal.pbio.3001437)
Supplement: Attachment — Submitted filename: Pinna et al PLOS Biol response to reviewers final.docx [file pbio.3001437.s005.docx]

**DEPARTMENT OF GENETICS, EVOLUTION AND ENVIRONMENT**

**UNIVERSITY COLLEGE LONDON**

**A prebiotic basis for ATP as the universal energy currency**

Silvana Pinna^1^, Cäcilia Kunz^1^, Aaron Halpern^1^, Stuart A. Harrison^1^, Sean F. Jordan^1^, John Ward^2^, Finn Werner^3^ and Nick Lane^1*^.

We have addressed the comments of the reviewers below. The reviewers’ comments are in Roman text and our answers are in red italics. We thank all three remaining referees for their enormous time, effort and expertise in reviewing our paper.

**REVIEWERS' COMMENTS:**

**Reviewer #1:**

[new comments, indicated by "R1':" are interdigitated]

R1: The manuscript by Pinna et al. reports on the detailed characterization of the phosphorylation of ADP by acetyl phosphate (AcP) to form ATP in the presence of ferric ions. This previously observed reaction appears to be surprisingly specific as no other NDPs or transition metal ions can carry out equivalent reactions to any significant extent. ADP phosphorylation appears to be optimal at 30°C, mildly acidic pH and low salt. The authors provide explanations for their observations within the context of their already well-established hypothesis of life's origins in a hydrothermal vent. Besides the fact that, taken face value, the observations reported here would rather favor a surface pond or lake as the cradle of life, some of their propositions need to be further substantiated.

R: We thank R1 for this clear synopsis. We agree that taken at face value our observations might seem to favour freshwater environments and we make this clearer in the revised version (p14 lines 401-408). We have also revised the order of the Discussion to bring up this passage before the section on work, as the paragraphs on work may have obscured this point in the original version.

R1': OK.

*Thank you.*

R1: Page 3 "ATP in a prebiotic, monomeric world". This assumes that life originated in bulk water. But, if, instead, life first appeared on mineral surfaces then the needed energy could have been provided through the oxidation of H2 and CO by, for instance, catalytic FeNiS minerals. Only when more evolved protocells liberated themselves from those surfaces, and moved to bulk water, would have ATP become essential.

R: We have revised the Introduction to make our reasoning clear here. We agree with R1 that the earliest forms of metabolism are indeed exergonic and could have occurred on mineral surfaces. But it is unlikely that this could account for purine nucleotide synthesis, for example, which has six phosphorylation steps. We lay out this perspective in the revised Introduction, specifically on pages 3-4, lines 69-83.

R1': In fact, there is no a priori reason to conclude that purine nucleotide synthesis could not have taken place on a mineral surface. The authors should check the paper by Deiana et al.  (ChemCatChem 2013, 5, 2832-2834.) who argue that "… the interactions of carboxylate oxygen atoms with surface Ti4+ ions, which act as Lewis acid centers, is expected to withdraw electron density from the C atom, which becomes electrophilic enough to undergo nucleophilic attack by the nitrogen atom of the amine". This is exactly the effect of phosphorylation on the carbonyl carbons of reaction intermediates, which allows for the formation of 9 C-N bonds during purine synthesis. Although acetyl phosphate could have been involved in ATP synthesis other pathways are also possible (see Akouche et al. Angew. Chem. Int. Ed. 2017, 56, 7920-7923 for the remarkable one-pot synthesis of AMP from phosphate, adenine, and ribose on a fumed silica surface).

*R1 is here responding to our comment above rather than the MS changes, which lay out our reasoning for considering acetyl phosphate in relation to purine synthesis at all. Having briefly laid out our reasons for using life as a guide to the origins of metabolism, we explicitly write:*

*“The idea that ATP could have arisen as a product of protometabolism starting from H2 and CO2 is therefore not unreasonable.”*

*We propose to add the following bold text to clarify our argument* ***(though we do not dispute that other mechanisms could potentially also give rise to purine nucleotides [REFS).***

*(We cite the two papers requested by R1 here). We continue:*

*“Nonetheless,* ***biological*** *purine synthesis specifically involves six phosphorylation steps that are catalysed by ATP in modern cells – an autocatalytic feedback loop.”*

*Finally, we note that we are clear that in our argument we are referring explicitly to the prediction emerging from using life as a guide to the origins of metabolism:*

*“****If ATP was indeed formed in a monomer word via a biomimetic protometabolism,*** *then an earlier ATP-equivalent must have driven the phosphorylation steps in purine synthesis. Why this earlier phosphorylating agent was replaced, and specifically with ATP rather than other nucleoside triphosphates, might explain why ATP later rose to prominence in metabolism.”*

*These changes make it clear that we are proposing a specific hypothesis about the synthesis of ATP following life as a guide to the origins of metabolism, and are testing one aspect of this hypothesis in the paper. At no point do we say that it is the only way in which this could have occurred, but we are not writing a review of the whole field so we believe these modifications make clear that we are testing a specific hypothesis in this paper.*

R1: "Even if only one nucleotide triphosphate can be dominant, the implication of a frozen accident is not a satisfying explanation." Why not? There are different levels of "frozen accidents". This has been discussed by Brandon Carter who, in 1984, proposed that the evolution towards intelligent life required 6 "hard steps", the first one being abiogenesis. Each step was statistically postulated to last between 600 and 800 million years. Although these periods of time may be considered workable in astronomy and geology, in biology they do not make the same sense. In the latter case it is necessary to invoke the occurrence of extremely unlikely (highly contingent) events to explain that time duration. They can be indeed defined as "frozen accidents".

R: We do not agree with R1 about frozen accidents, but this is a relatively trivial point in relation to the paper. We have de-emphasised frozen accidents in the revised Introduction, and instead stressed the centrality of other adenine nucleotide cofactors including NAD, FAD and coenzyme A, all of which point to the importance (and so availability) of adenosine or ATP in early metabolism.

R1': As the authors point out there is no doubt that adenine nucleotide must have been around quite early. A "frozen accident" in this context describes the -most likely- very low global probability for the required reactants to find themselves in the same location under productive conditions. Once that accident happens subsequent evolution will propagate the result in both time and space if it confers an advantage.

*We disagree with R1 about this, but again we have been careful with our choice of words in the revised MS. R1 writes that ‘subsequent evolution will propagate the result in both time and space’ but we would respectfully disagree with this statement. What exactly is being selected and how? This is the explicit subject of three of our recent papers (see most recently Harrison et al BBA Bioenergetics, 1863: 148597 (2022), which provide a detailed framework for how nucleotide synthesis could potentially occur in protocells subject to selection. We have thought long and hard about the possible units of selection before the origin of the genetic code.*

*R1 is perfectly entitled to disagree with our perspective, and might be right, but nonetheless we are presenting the perspective that drove us to undertake the work, and which is consistent with multiple other papers of ours taking an explicitly evolutionary approach to the origin of life. However, the Introduction to the paper is not the place to discuss these ideas.*

*We have therefore modified the text simply to be clear that this is our own view:*

**In our view,** the prominence of ATP is unlikely to reflect a frozen accident, as adenine nucleotide cofactors are also ubiquitous across intermediary metabolism, including the ancient cofactors NADH, FADH and coenzyme A.

R1: Page 13. "Such steep pH gradients could in principle operate across protocells as well as inorganic barriers." The authors propose that ATP would have been synthesized outside protocells, under acidic conditions, to be then transported across a proto-membrane into a cell where, under alkaline conditions, it would have phosphorylated some substrate(s). Have they considered the implications of such a mechanism in terms of ATP/ADP concentrations and diffusion? ATP would have to diffuse towards the protocell and ADP do it in the opposite direction, without risking to disappear into the bulk water. Another crucial point is membrane permeability. As F. H. Westheimer wrote in 1987: "Phosphoric acid is specially adapted for its role in nucleic acids because it can link two nucleotides and still ionize; the resulting negative charge serves both to stabilize the diesters against hydrolysis and to retain the molecules within a lipid membrane. A similar explanation for stability and retention also holds for phosphates that are intermediary metabolites and for phosphates that serve as energy sources." So, one very important role of even proto-membranes should have been to keep charged metabolites inside the cell to sustain metabolism. This, in turn, would have made ADP diffusion out of the protocell difficult to envision.

R: This is a complete misunderstanding of what we had attempted to write, and we can only apologize for not being sufficiently clear. We agree with R1 (and Westheimer's classic paper) about phosphorylated intermediates, including especially triphosphates, not being able to cross cell membranes, even simple fatty acid bilayers. In fact, we envisaged ADP being phosphorylated inside the protocells immediately adjacent to the membrane, subject to proton influx from the acidic exterior. No phosphates ever cross the membrane in our model. We have made this clear in the revised Discussion (page 15, lines 435-443).

R1': I do not think that 'misunderstanding' is the right way to describe the problem in the original text (p 13). There, the authors wrote "…that thin inorganic barriers … can sustain proton gradients…". And later they concluded "… could promote the phosphorylation of ADP to ATP under locally acidic conditions close to the barriers, followed by hydrolysis linked to phosphorylation under more alkaline conditions in the cytosol of protocells". The question is, where are the "locally acidic conditions close to the barriers"? In the cytosol, as well? So, is there an intracellular gradient? This would be quite different from standard biological proton gradients.

The corrected text is fine.

*Thank you. The point is that we had completely failed to convey what we had intended, and we are pleased that R1 is satisfied with the revised text.*

R1: Page 15. "…enabling ATP to drive work even in a prebiotic monomer world." How are the authors defining 'work' in that setting? In contemporary biology work is i) performed mechanically as in muscle contraction and ii) against a gradient of chemical potential in active transport (see Jencks, 1989). This work depends on complex protein-based binding energy modulation and the coupling of intermediate reaction steps. Of course, this kind of work could not have existed prebiotically. At that time the most likely role of ATP (if it already existed) would have been, for instance, phosphorylation to render carbonyl carbons electrophilic enough to be attacked by a nucleophile such as ammonia. It is debatable that this process would qualify as work.

R: Our perspective is grounded in the idea of a monomer world, and we do not envisage any protein-based machinery here at all. We have made this clearer at several points in the revised MS and added a new paragraph to the end of the Discussion (page 15 lines 451- 464) where we discuss specifically what we mean by work in this context (essentially coupling exergonic to endergonic reactions, and in particular the polymerization of nucleotides and amino acids).

R1': OK

*Thank you.*

R1: Page 31. Figure 6. Here and in the text, it is implied that the N6 -NH2 group interacts with Fe3+. In A-T base pairing N6 interacts with the O atom from the carbonyl group of T through a polarized + proton. What would be the driving force in its postulated interaction with Fe3+? In b, the metal ion is hexacoordinated but the coordination geometry is not clear. In any case, it doesn't look octahedral.  Maybe the authors should do some modeling to check whether the proposed arrangement is plausible. An experimental confirmation of their model would be even better.

R: We thank R1 for pointing out that base pairing in DNA and agree that our model as originally depicted was incorrect. We have also undertaken a series of molecular dynamic simulations, as suggested by R1, which do indeed strengthen our mechanistic interpretations. We discuss these new MD simulations at length in the Results section on page 10, lines 259-288, in two new figures (Fig. 6 and SI Fig. 7), and in the Discussion on page 12 lines 330-337. We use this new information to modify our proposed mechanism, specifically in relation to the N6 amine group and the N7, which is presented in Fig. 7. These new sections also respond to the concerns of other reviewers.

R1': I think there is a problem with the overall charges. In Fig. 7a ADP carries 3 negative charges, one on its alpha phosphate and two on its beta phosphate. In fact, one of the O- of the beta phosphate is the nucleophile that initiates ATP synthesis. However, in both the section about MD simulations and the legends of Figs. 6 and 7, ADP is described as having a 2- valence. Because the MD result is based on the electrostatic properties of the reactants one negative charge difference should have a major impact. This has to be clarified and revised.

In Fig. 7b the ferric ion might be tetrahedral but, again, the coordination sphere is not well defined. It may be out of the scope of this paper, but better modeling would come out of a QM/MM analysis of the proposed structures.

*We explain at the top of page 11 that the proximity of Fe^3+^ leads to deprotonation of the outermost OH group:*

*“The interaction with the dianion has been proposed before [83,84] and is key because at the optimal pH of 5.5–6, the first two hydroxyl groups of ADP (pKa 0.9 and 2.8) are deprotonated, while the external OH group (pKa 6.8) remains protonated, and is therefore not available for nucleophilic attack [85]. The interaction of the two deprotonated OH groups with Fe3+ has the effect of lowering the pKa of the outermost OH group, thus deprotonating it and enhancing its nucleophilicity (****Fig. 6b****).”*

*We have added in a note on page 20 to say:*

*“but as noted previously, the proximity of Fe^3+^ lowers the pKa of the outermost OH group, resulting in* its deprotonation and increasing its nucleophilicity. We did not explicitly model this in the MD simulations as this would require quantum simulations of the reactions themselves”.

In relation to the coordination sphere, we have added a note to the figure legend to clarify:

*“Note that we only depict interactions between between the Fe^3+^ and moieties on the ADP, adenosine ring and AcP that have been established by our experiments and MD simulations, and this is not intended as a full depiction of the coordination sphere; for clarity we do not include any interactions with water which would certainly be part of the coordination sphere.”*

R: We thank R1 again for their helpful comments and hope that we have addressed them to their satisfaction. The revisions very clearly improve the paper.

R1': You are welcome and please fix the remaining problems.

*We have done so. Thank you again for your invaluable input.*

**Reviewer #3:**

The authors have made considerable, significant and positive changes to their original submission. It is clear from the combined reviewers comments that many different features of this paper have benefitted from these changes.

From the perspective of this reviewer in particular, (i) the mechanistic aspects of ADP phosphorylation via AcP in the presence of Fe3+ has now been nuanced significantly, in line with accepted chemical precedence. (ii) Discussions around the "value" of mild, non-enzymatic ADP phosphorylation to a burgeoning prebiotic "organism" have also been modified and some additional caution introduced.

The question as to how, where and when nucleotide phosphorylation became a fundamental component of primitive biological organisms is a foundational one in origin-of-life studies and its discussion is relevant to the current paper. However, the experimental offerings here are not focused on addressing that question, but rather on questions of chemistry. In this regard, I'm happy that the authors have sufficiently strengthened these chemical features, whilst modulating the language around potential "value" judgements which are less well substantiated.

*We completely agree that the paper has benefited greatly from the detailed comments of the reviewers and we thank all four for their detailed comments. We thank R3 specifically for their constructive attitude at this point to seeing the paper published.*

**Reviewer #4:**

The authors have taken into account prior recommendations and made changes as needed.  Though there are different perspectives on this time of history (whether life was autotrophic early on is an open question), the authors have approached the subject reasonably.

*We agree with this statement on perspectives, and are very pleased that R4 sees our approach as reasonable in the context. We sincerely thank R4 for their extremely valuable and constructive comments.*
